# Cue-exposure treatment influences resting-state functional connectivity—a randomized controlled fMRI study in alcohol use disorder

**DOI:** 10.1007/s00213-024-06531-x

**Published:** 2024-01-23

**Authors:** Àlvar Farré-Colomés, Haoye Tan, Sarah Gerhardt, Martin Fungisai Gerchen, Martina Kirsch, Sabine Hoffmann, Peter Kirsch, Falk Kiefer, Sabine Vollstädt-Klein

**Affiliations:** 1 Department of Addictive Behaviour and Addiction Medicine, Central Institute of Mental Health, Medical Faculty of Mannheim, Heidelberg University, 68159 Mannheim, Germany; 2 Department of Clinical Psychology, Central Institute of Mental Health, Medical Faculty of Mannheim, Heidelberg University, 68159 Mannheim, Germany; 3 Bernstein Center for Computational Neuroscience Heidelberg/Mannheim, 68159 Mannheim, Germany; 4 Department of Psychology, Heidelberg University, 69117 Heidelberg, Germany; 5 Mannheim Center for Translational Neurosciences (MCTN), Medical Faculty of Mannheim, Heidelberg University, 68159 Mannheim, Germany; 6 Feuerlein Center on Translational Addiction Medicine, Heidelberg University, 69117 Heidelberg, Germany

**Keywords:** Alcohol use disorder, Cue exposure treatment, fMRI, Resting-state, Default mode network, Salience network, Dorsal attention network, Insula, Striatum

## Abstract

**Rationale:**

Cue-exposure therapy (CET) consists of exposing patients to the cause of their affliction in a controlled environment and after psychological preparation. Ever since it was conceived, it has been suggested as a treatment for different types of behavioural impairments, from anxiety disorders to substance abuse. In the field of addictive behaviour, many different findings have been shown regarding the effectiveness of this therapy.

**Objectives:**

This study aims to examine the underlying neurobiological mechanisms of the effects of CET in patients with alcohol use disorder using resting-state functional magnetic resonance imaging (rs-fMRI).

**Methods:**

In a randomized, controlled study, we examined patients after inpatient detoxification as well as healthy controls. Patients underwent nine sessions of CET spaced over 3 weeks. Rs-fMRI was conducted before treatment and 3 weeks after treatment onset in patients, healthy controls received only one rs-fMRI measurement. The final participant sample with complete data included 35 patients in the CET group, 17 patients in the treatment-as-usual group, and 43 HCs.

**Results:**

Our results show differences in the Salience Network when comparing the CET group to the treatment-as-usual group (TAU). Functional connectivity between the anterior cingulate Cortex (ACC) and the insula was increased after CET, whereas it was decreased from ACC to the putamen and globus pallidus. Further, increased connectivity with the precuneus was found in the dorsal attention network after cue exposure treatment.

**Conclusions:**

These findings suggest that cue exposure therapy changes the resting-state brain connectivity with additional effects to the standard psychotherapy treatment. Hence, our study results suggest why including CET in standard therapies might improve the preparation of patients in front of daily situations.

**Supplementary Information:**

The online version contains supplementary material available at 10.1007/s00213-024-06531-x.

## Introduction

Alcohol use disorder (AUD) is a disorder often characterized by impulsive and compulsive behaviour. Alterations of resting-state networks in individuals with AUD have been widely studied. In general, the resting brain of these patients shows network-specific patterns of aberrant functional connectivity that have been related to different emotional and behavioural outcomes (Müller-Oehring et al. 2015). The main resting-state networks affected in AUD are the default mode network (DMN) and the salience network (SN). A within-network connectivity deterioration was mainly reported in both, and also, a larger outside-network connectivity has been suggested as a compensation mechanism (Chanraud et al. 2011; Müller-Oehring et al. 2015; Sullivan et al. 2013).

The main nodes of these networks are brain regions that are known to be affected and deteriorated by alcohol abuse. Areas like the anterior cingulate cortex (ACC) or the insula, parts of the SN, have been reported to show aberrant metabolism and/or functional connectivity (Goldstein and Volkow 2011; Müller-Oehring et al. 2015; Sullivan et al. 2013). Moreover, some brain regions altered in alcohol abuse disorder are the main nodes of the DMN. The posterior cingulate cortex (PCC) and the medial prefrontal cortex (mPFC) have been reported to present enhanced functional connectivity in AUD individuals (Chanraud et al. 2011; Müller-Oehring et al. 2015). Further, frontostriatal functional connectivity was shown to be associated with alcohol-related variables like craving or self-efficacy to abstain from alcohol (Gerchen et al. 2019; Gerchen et al. 2021).

The dorsal attention network (DAN) is involved in goal-directed, voluntary control of visuospatial attention (Corbetta and Shulman 2002; Kincade et al. 2005). In a previous study, this network showed increased connectivity with the somatosensory cortex in AUD patients (Farré-Colomés et al. 2021). Its dynamic interaction with the SN (Vossel et al. 2014) is very interesting in terms of saliency processing and cue-reactivity, key features of AUD.

In the past years, the study of AUD has increasingly focused on cue-reactivity. This phenomenon is described as the psychological or physiological response that results from exposure to alcohol-related stimuli. Considering the current situation of alcohol in our society, this component of alcoholism can be decisive in maintaining the abstinence of patients and improving their quality of life within our society. So often, AUD individuals find cues in the environment that relate to alcohol use, and it facilitates relapse significantly (Conlkin and Tiffany 2002; Schacht et al. 2013). Cue exposure in our society is a very serious problem for patients who seek rehabilitation (Yalachkov et al. 2012). Cue-reactivity is the direct result of cue exposure. It can be described as a learned intrinsic response to some environmental cues triggering specific neuronal responses. After a long association period, this learning process is considered to end up with addictive behaviour (Drummond 2000; Everitt and Robbins 2005; Mellentin et al. 2017). Learning theory states that addiction is a learning process achieved by reinforcement mechanisms. For this reason, it is considered that cue reactivity can be suppressed or weakened by new learning based on exposing relevant drug cues but deleting the habitual behaviour (drug use).

Alcohol-related cues are known to activate the putamen, the ACC and the mPFC, the Orbitofrontal Cortex (OFC), the Striatum, and many other areas in AUD patients (Courtney et al. 2016; Grüsser et al. 2004; Heinz et al. 2009; Vollstädt-Klein et al. 2010), all being important due to their connection with the insula (Grüsser et al. 2004; Menon and Uddin 2010). Daily exposition to alcohol cues triggers responses related to neural processes linked to craving and alcohol drinking in AUD patients (Miranda Jr. et al. 2020; Monti et al. 2000). Still, these outcomes derived from cue exposure seem to be sensitive to medication in both treatment seekers and non-treatment seekers and in adults and adolescents (Miranda Jr. et al. 2020). In this context, cue-exposure treatment (CET), sometimes referred to as cue-extinction training or cue-exposure therapy, is based on repeated exposure to environmental stimuli that were previously associated with drug use in order to decrease the inner response of the patient in front of these cues (Conlkin and Tiffany 2002). Thus, the final aim of this procedure is to eliminate learned responses to drug cues using repeated non-reinforced exposure (Mellentin et al. 2017).

Previous studies report how patients who received the treatment as usual with CET in addition show reduced cue-elicited activation in the striatum, the ACC, the dorsal prefrontal cortex (dPFC), the insula, and the IFG (Courtney et al. 2016; Kiefer et al. 2015; Vollstädt-Klein et al. 2010). These studies suggest that CET may improve the response of neural substrates in conditioned cue reactivity, despite not having demonstrated strong effects on substance use itself (Conlkin and Tiffany 2002). Small improvements have been linked to CET in a review by Mellentin et al. (2017) regarding the final drinking intensity, frequency, total drinking score, and latency to relapse. However, no studies have focused on the effects of CET on resting-state functional connectivity.

In this study, we aimed to characterize the changes in the resting brain that CET can promote after a period of training in abstinent, treatment-seeking patients with AUD. To our knowledge, this approach is being used for the first time, which might improve knowledge of the CET effect in AUD patients. The analysis of resting-state connectivity patterns may help to understand if there is an actual change in brain connectivity and, moreover, be in line with the findings by Vollstädt-Klein et al. (2011) where reduced neural cue-reactivity was reported after CET. Based on previous studies (Müller-Oehring et al. 2015), we hypothesised that resting-state connectivity would change in patients after a 3-week CET program compared to patients with usual treatment only. Within the SN, resting-state functional connectivity of the ACC was hypothesised to be decreased towards striatal regions and increased towards the inferior parietal lobe and the precuneus in patients with CET. In the DAN, we hypothesised a reduction in functional connectivity between the FEF and motor areas of the cortex, described in a previous study (Farré-Colomés et al. 2021). As a change in the DMN, we expected an increase in frontal cortex functional connectivity with the cingulate cortex, as compensation for the AUD effects on this network.

## Materials and methods

### Participants

**Fig. 1:**
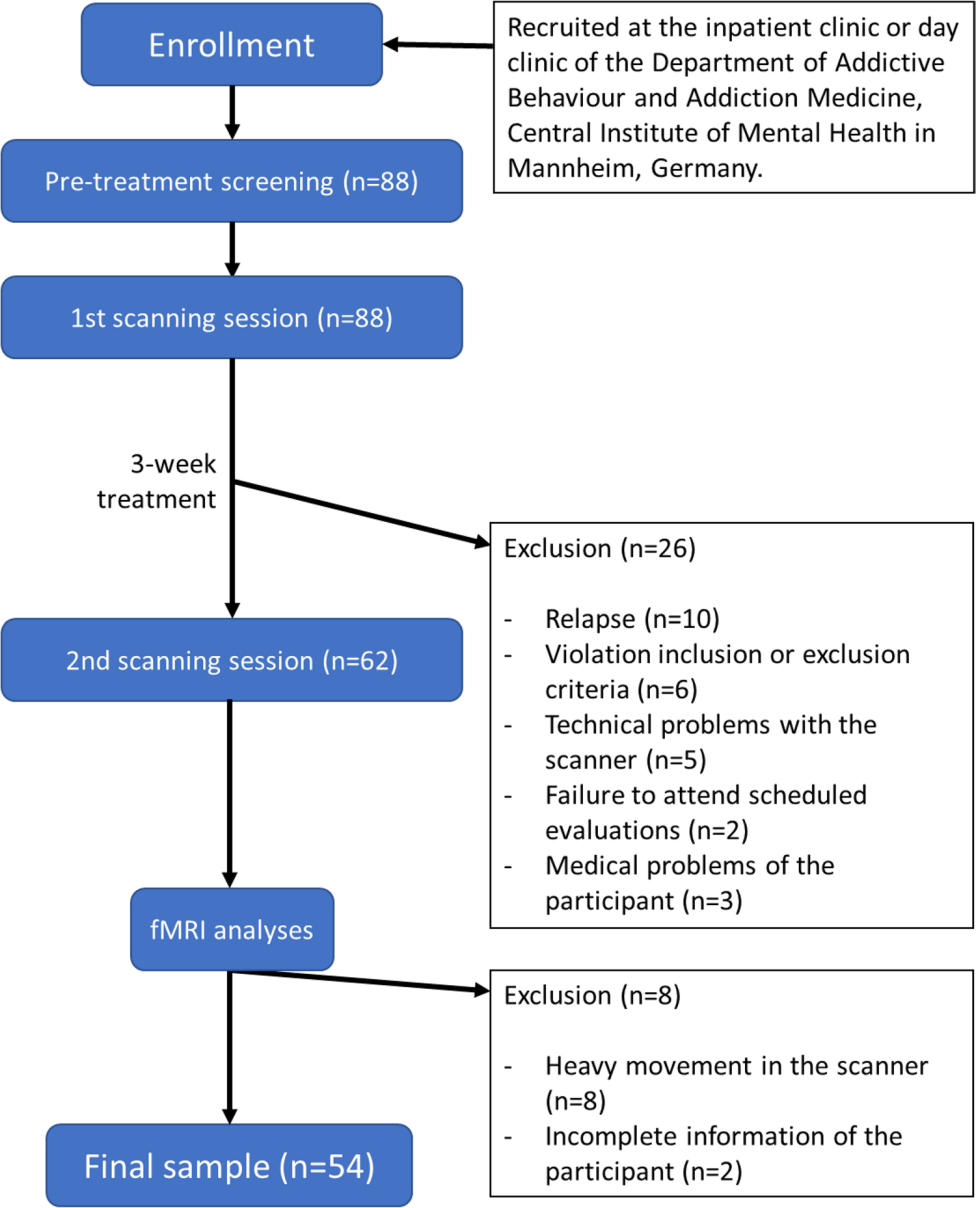
CONSORT flowchart. Description of the study design and participant drop-out reasons

Study participants with AUD were recruited in the Department of Addictive Behaviour and Addiction Medicine, Central Institute of Mental Health in Mannheim, Germany (Deutsches Register Klinischer Studien (German Clinical Trials Registry) ID: DRKS00003388). Suitable participants were selected after undergoing a pre-treatment screening and were diagnosed with AUD according to the Diagnostic and Statistical Manual of Mental Disorders (DSM-5, assessed with Structured Clinical Interview (SCID-I) for DSM-IV (Wittchen et al. 1997) due to the unavailability of a SCID for DSM-5 at the time of examination). They did not present other axis I psychiatric disorders or substance abuse (except for nicotine). Patients were abstinent from alcohol on an average of 11 days ± 5.6 (standard deviation), with a range of 5 to 31 abstinence days. The severity of alcohol dependence was assessed using the Alcohol Dependence Scale (ADS) (Skinner and Horn 1984). The age range included participants between 18 and 65 years old at the time of testing. Using a randomization function (2:1), the selected participants were randomly distributed into two groups, the Treatment-As-Usual (TAU) group and the Cue Exposure Treatment (CET) group. Of the initial 84 selected patients, 26 did not undergo the second scanning session (Fig. 1). The study was completed by 58 patients with 39 participants in the CET group and 19 participants in the TAU group. From the CET group, 4 subjects were excluded after checking the quality of the images. In the TAU group, 2 subjects were excluded after the quality check. A total of 45 healthy controls (HC) were recruited from the community by advertisements, of whom 2 were excluded after a quality check. All participants were granted written informed consent to participate in the study. The study protocol was approved by the ethics committee of the Medical Faculty Mannheim at the University of Heidelberg (2011-303N-MA). Questionnaire scores and characteristics of the analysed sample, with respect to the different groups, are presented in Table 1.

**Table 1:** Mean (SD) group demographic data and questionnaire scores of all participants (*N* = 95)

	TAU	CET	HC	Test statistics
*N*	17	35	43	
Gender [male to female]	16:1	25:10	26:17	*χ*^2^(2) = 6.65, *p* = 0.036*
Age	50.71 (9.39)	46.74 (9.12)	46.63 (9.56)	*F*(2,92) = 1.29, *p* = 0.28
Smoker [yes/no]	9:8^1^	24:11^2^	4:39^1,2^	*χ*^2^(2) = 30.21, *p* = 0.000*
Abstinence days	17.29 (16.25)^1^	19.25 (25.54)^2^	78.83 (12.53)^1,2^	*F*(2,88) = 107.36, *P* = 0.000*
ADS	13.68 (6.11)^1^	14.82 (6.05)^2^	1.73 (1.65)^1,2^	*F*(2,88) = 84.09, *p* = 0.000*
AUQ				
Pre-treatment	13.22 (4.89)^1^	13.02 (5.32)^2^	9.52 (2.82)^1,2^	*F*(2,97) = 8.45, *p*= 0.000*
Post-treatment	12.70 (8.74)	10.85 (4.16)	−	*t*(49) = 1.98, *p* = 0.165
Change from pre- to post-treatment^i^	−0.82 (9.15)	−2.36 (6.34)	−	*F*(1,48) = 0.32, *p* = 0.573
OCDS				
Pre-treatment	16.15 (7.37)^1^	15.97 (6.29)^2^	1.31 (1.55)^1,2^	*F*(2,100) = 106.00, *p* = 0.000*
Post-treatment	11.11 (5.50)	9.75 (5.63)	−	*t*(52) = 0.07, *p* = 0.793
Change from pre- to post-treatment^i^	−4.94 (7.05)	−6.05 (6.66)	−	*F*(1,52) = 0.013, *p* = 0.909
AASE V				
Pre-treatment	30.89 (18.22)^1^	30.50 (18.10)^2^	7.62 (11.49)^1,2^	*F*(2,98) = 26.71, *p* = 0.000*
Post-treatment	19.21 (16.48)	13.52 (12.80)	−	*t*(52) = 2.61, *p* = 0.112
Change from pre- to post-treatment^i^	−11.67 (19.37)	−17.27 (15.72)	−	*F*(1,52) = 34.77, *p* = 0.259
AASE Z				
Pre-treatment	52.93 (24.68)^1^	59.64 (17.60)^2^	68.37 (20.67)^1,2^	*F*(2,97) = 4.25, *p* = 0.017*
Post-treatment	61.20 (21.66)	64.97 (18.78)	−	*t*(50) = 0.30, *p* = 0.522
Change from pre- to post-treatment^i^	6.85 (29.50)	6.18 (12.64)	−	*F*(1,48) = 0.01, *p* = 0.911
Cue-induced Craving^†^				
Pre-treatment	0.26 (1.85)	−0.58 (8.49)	−	*t*(56) = 2.48, *p* = 0.558
Post-treatment	0.55 (2.89)	−0.07 (0.42)	−	*t*(54) = 15.28, *p* = 0.188
Change from pre- to post-treatment^i^	0.27 (2.90)	0.52 (8.54)	−	*F*(1,54) = 0.01, *p* = 0.905

*ADS* alcohol dependence scale, *AUQ* alcohol urge questionnaire, *OCDS* obsessive compulsive drinking scale pre- and post-treatment scores, *AASE V* alcohol abstinence self-efficacy scale, temptation to drink, *AASE Z* alcohol abstinence self-efficacy scale, self-efficacy to abstain

^*^Significant post-hoc results (*p* < 0.05)

^†^Values measured on a visual analogue scale (ranging from 0 to 10)

^1,2,3^With respect to the group—Chi2 for dichotomous variables, ANOVA for 3 groups, *t*-test for 2 groups, and repeated measures ANOVA for the interaction of group × timepoint (group comparison of changes from pre- to post-treatment)

^i^Results of the interaction between group and time

### Study design

This study was part of a larger project. The original sample size estimation was performed related to the main outcome of tasked-based fMRI, and these results are published elsewhere (Becker et al. 2018; Kirsch et al. 2015). In the current article, we report exploratory resting-state analyses from this project.

Before engaging in the study, participants with AUD had to overcome controlled abstinence for a period of 5 to 21 days under medical supervision until no severe alcohol withdrawal symptoms were present. Treatment-as-Usual (TAU) consisted of a qualified alcohol detoxification treatment at the clinic for 3 weeks. It consisted of health education, psychotherapeutic treatment in single and group sessions, competence training, relaxation exercises, sports programs, occupational therapy, and sociotherapy. Participants that were included in the CET group received 5 to 9 standardized individual CET sessions (mean 6.86, SD 0.96) additionally to TAU. These sessions followed the guidelines of a validated treatment manual (Mann 2006) that has been previously applied in other studies (Kiefer et al. 2015; Vollstädt-Klein et al. 2011). AUD participants were scanned before and after the treatment (Fig. 1). Moreover, the Obsessive Compulsive Drinking Scale (OCDS) (Anton 2000) and the Alcohol Abstinence Self-Efficacy Scale (AASE) (DiClemente et al. 1994) were fulfilled also before and after the 3-week treatment to assess the severity of alcohol craving. For the CET training sessions, it was asked to the participant to imagine critical drinking situations. Then the participant was exposed to the favourite alcoholic drink. Participants were informed in the first session about the drug-associated reactions this exposure could evoke in them, but also that this is expected to improve their reaction over time during the sessions. Besides, they were informed that CET sessions would continue until they stop feeling the craving. Participants then had to set up a hierarchy of situations that could trigger a relapse. In the following sessions, patients had to imagine one of these critical situations before being exposed to their favourite drink. They were instructed to handle the alcohol bottle, pour a drink, and smell the drink without consuming it. Meanwhile, participants had to be informed several times about the craving-related feelings, cognition, and physical reactions. Patients were asked not to stop focusing their attention on the situation they had imagined. The CET training sessions lasted between 30 and 90 min and ended when the participant stopped feeling any craving (Mann 2006). HC participants were only scanned once and underwent the same assessment as the AUD participants at the first session.

### fMRI data acquisition

Functional imaging data was obtained by using a 3 T whole-body tomography Siemens Magnetom Trio Scanner (Siemens Medical Systems, Erlangen, Germany). Participants were asked to close their eyes during the 9 min of resting-state acquisition. They were also instructed to keep thoughts flowing, to not think of anything in particular, and to avoid attaching to any thought. High-resolution 3-dimensional T1-weighted anatomical images (MPRAGE) were collected to evaluate individual brain morphology (repetition time (TR) = 2.3 s, eco time (TE) = 3.03 ms, flip angle = 9°, field of view (FOV) = 256 × 256 mm^2^, slice thickness = 1 mm, voxel dimensions = 1 × 1 × 1 mm^3^, matrix size = 256 × 256, and 192 sagittal slices). For the measurement of the blood oxygen level-dependent (BOLD) contrast, 267 T2*-weighted echo-planar images (EPI) (TR = 2 s, TE = 30 ms, flip angle = 80°, 28 transversal slices, slice thickness = 4 mm, 1 mm gap, voxel dimensions = 3 × 3 × 3 mm^3^, FOV = 192 × 192 mm^2^, and matrix size = 64 × 64) were acquired in descending order.

### fMRI preprocessing

Imaging data were processed using the CONN toolbox v20b (Whitfield-Gabrieli and Nieto-Castanon 2012) performed in MATLAB 2020a through SPM12 (Wellcome Department of Imaging Neuroscience, London, UK). The default pipeline from the CONN toolbox was used for the preprocessing of structural and functional images. It is composed of several steps: realignment and unwarping (with subject motion estimation and correction), centering, slice-time correction, outlier detection with ART-based (Artefact Detection Tools) identification for scrubbing with conservative parameters (95th percentiles, *z*-value threshold of 3 in global-signal and 0.5 mm subject-motion threshold), segmentation (white and grey matter, cerebrospinal fluid), normalization (Montreal Neurological Institute atlas), and smoothing at 8 mm full-width half maximum (FWHM) Gaussian kernel. Following the preprocessing steps, the blister variables provided by the CONN toolbox were regressed out of the signal (white matter, CSF, realignment, and scrubbing). Data were filtered using a 0.008–0.09 Hz band-pass and linear trends were suppressed using linear detrending. Individuals with excessive head movement (> 3 mm/3°) and/or other artefacts like signal drop out or incomplete field of view were discarded. Consequently, 8 subjects were discarded from the study.

### Connectivity analyses

**Fig. 2:**
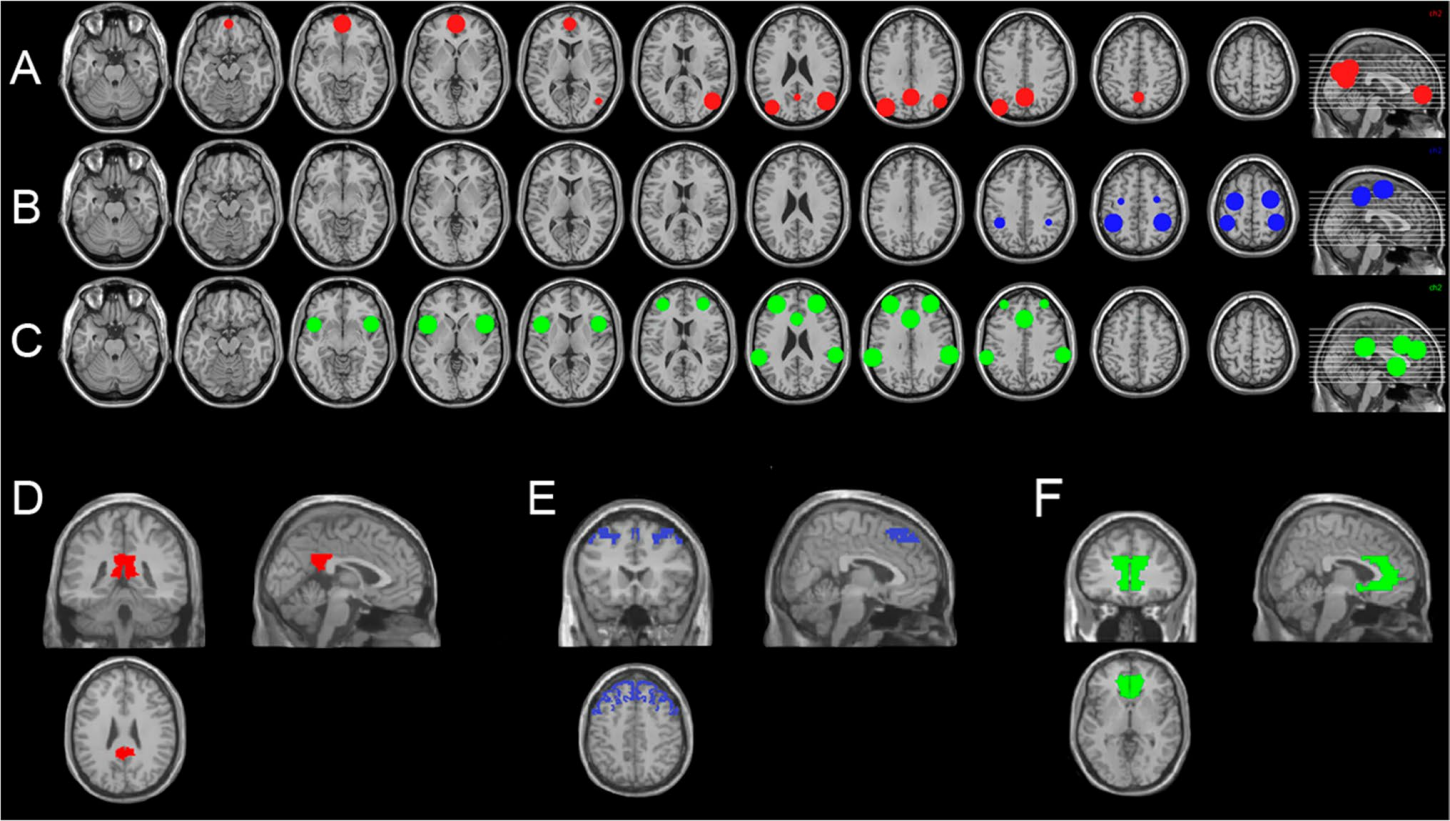
Representation of the examined resting-state networks and their corresponding nodes. **A** DMN display including the middle prefrontal cortex, the right and left angular gyri, and the posterior cingulate cortex. **B** DAN depiction including the right and left frontal eye fields and the right and left intraparietal sulcus. **C** SN representation including the anterior cingulate cortex, the right and left anterior insula, the right and left rostral prefrontal cortex, and the right and left supramarginal gyri. **D** Location of the posterior cingulate cortex (*x* = 89, *y* = 84, *z* = 100) used as a seed to examine the DMN. **E** Display of the right and left frontal eye fields (*x* = 98, y = 147, *z* = 123) used as a seed to examine the DAN. **F** Detail of the anterior cingulate cortex (*x* = 96, *y* = 160, *z* = 71) used as a seed to examine the SN

Second-level analyses were based on a seed-to-voxel first-level connectivity analysis. The CONN toolbox provides default seeds for each major network of the brain. After revision of the literature, we decided to use the DMN, the SN, and the DAN displayed in Fig. 2 (Chanraud et al. 2011; Corbetta and Shulman 2002; Kincade et al. 2005; Müller-Oehring et al. 2015; Sullivan et al. 2013; Vossel et al. 2014). To compare connectivity at rest, the toolbox uses the images generated in the first-level analyses. A seed-to-voxel approach was used to compare differential connectivity patterns in the resting brains of the different groups after treatment. Seeds were located by the CONN toolbox default parameters: the posterior cingulate cortex (PCC) was used for the DMN, the anterior cingulate cortex (ACC) for the SN, and the frontal eye fields (FEF) for the DAN. An introductory approach was conducted by comparing both AUD groups to the HC group. We analysed possible differences between groups regarding age, gender, and smoking status using a Chi^2^ test. Both treatment groups differed significantly in the gender of the participants. Further, the control group showed differences from both treatment groups in the smoking status. Therefore, we controlled for these variables (including them as covariates of no interest). All the data and statistics on demographic data can be found in Table 1.

Changes between pre-treatment and post-treatment were examined for each of the study groups (TAU, CET, and HC). Differences between the AUD participants and the HC are already described elsewhere (Gerchen et al. 2019; Gerchen et al. 2021) and were also included in the supplementary material. Hence, in this study, we report differences in post-treatment between HC, TAU, and CET groups. The probability of a family-wise error (FWE) was set to 0.05 to control for multiple statistical testing. For this purpose, we used the AlphaSim method implemented in the NeuroElf toolbox (www.neuroelf.net) using the 25000 Monte Carlo simulations with a voxel-level primary threshold of *p* < .005, resulting in a cluster-level extent threshold of *k* > 40 contiguous voxels. Smoothness estimation was conducted by SPM and based on residual images taking the maximum of the 3 estimated parameters in *x*, *y*, and *z* directions. We looked at the interaction between group and time and created ROIs as functional masks derived from significant interactions, i.e., differences in connectivity changes from pre-treatment (T1) to post-treatment (T2) between CET and TAU. Then we applied small volume correction (SVC) using these ROIs as masks when looking at the post-hoc comparison at T2 between CET vs TAU. The same statistical parameters were used to create the masks and to perform the SVC (*p* < 0.005 and *k* > 40).

## Results

The study sample included participants with moderate alcohol dependence according to the alcohol dependence scale (ADS). Alcohol urge (AUQ) in the treatment groups showed an initial drinking urge not much higher than the HC group. When considering the compulsive drinking scale (OCDS), both treatment groups presented intermediate levels at pre-treatment. The temptation to drink questionnaire (AASE-V) displayed intermediate temptation levels and did not present differences between treatment groups. However, the HC group showed a significantly lower temptation to drink compared to both treatment groups. It is of note that the CET group started with a slightly higher mean score in this questionnaire and displayed lower post-treatment mean scores, despite not being significant. In the craving pre-treatment, the TAU group reported significantly less craving than the CET group both before and after the scan. However, the CET reported that post-treatment had significantly lower craving levels than TAU after the scanning session.

**Fig. 3:**
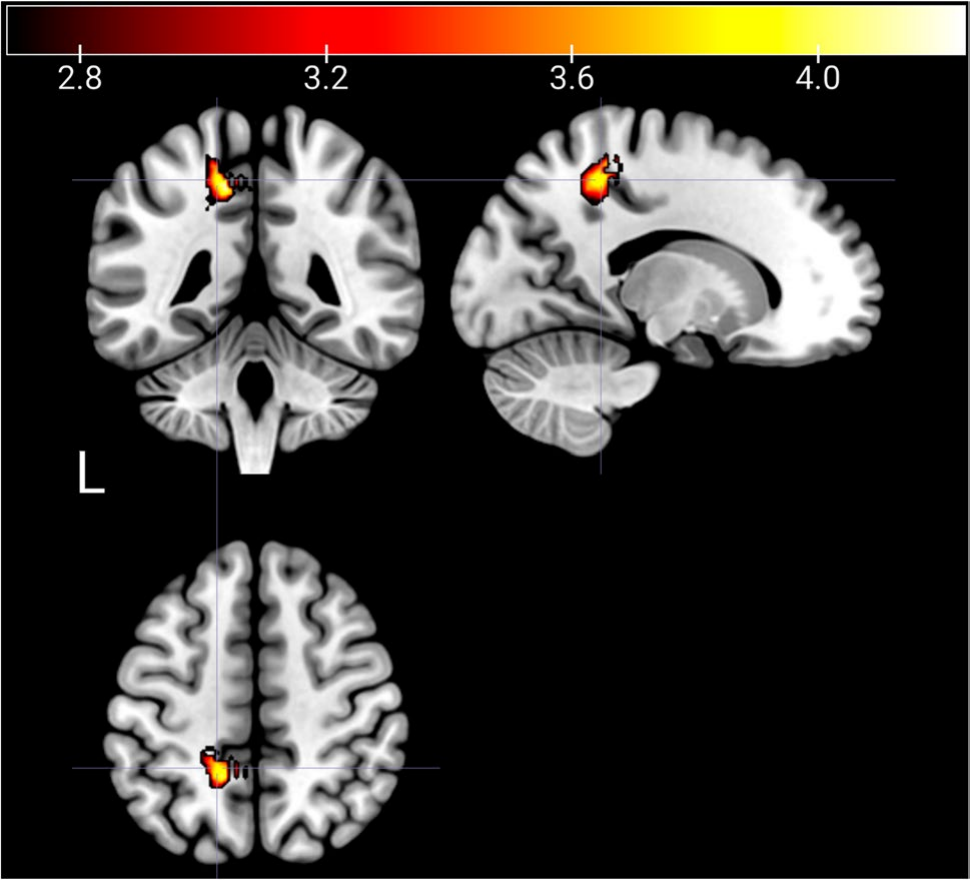
Increased resting-state BOLD connectivity within the DAN in CET compared to the TAU group after treatment. The image shows the increase of functional connectivity in the precuneus (*x* = −14, *y* = −46, *z* = 48) using FEF as a seed. *p*-level < 0.005; *k* > 40 voxel

**Fig. 4:**
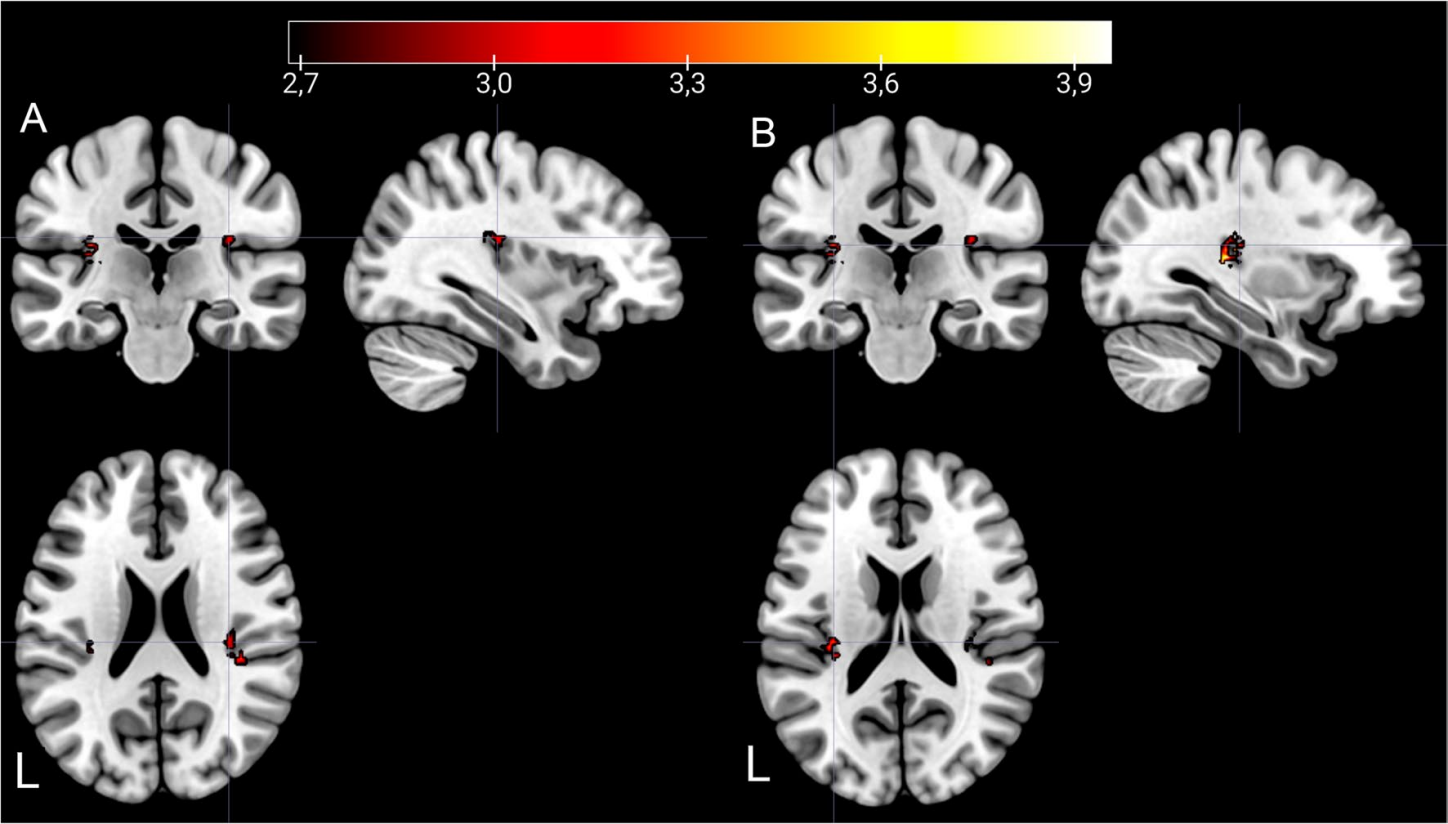
Increased bilateral alteration of resting-state BOLD connectivity within the SN in CET compared to the TAU group after treatment. The image shows an increase in functional connectivity in the insula (**A** *x* = 36, *y* = −30, z = 22; **B** *x* = −32, *y* = −30, *z* = 12). *p*-level < 0.005; *k* > 40 voxel

**Fig. 5:**
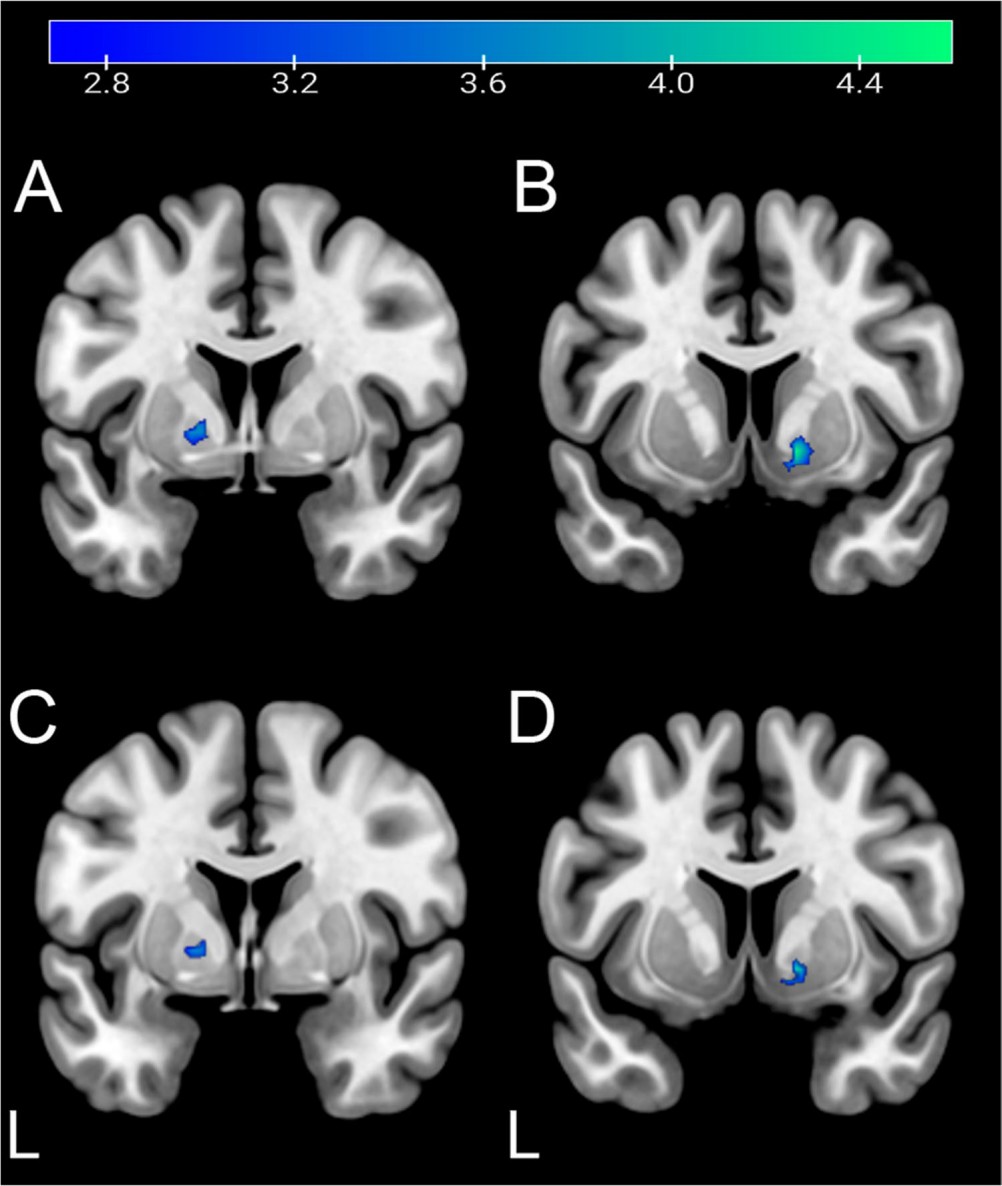
Increased bilateral alteration of resting-state BOLD connectivity within the SN in TAU compared to the CET group after treatment. The image shows a decrease in functional connectivity in the putamen (**A** *y* = 2; **B** *y* = 8; **C** *y* = 1; **D** *y* = 7). *p*-level < 0.005; *k* > 40 voxel

The resting-state fMRI analyses showed interaction effects between treatment and time. The SN and the DAN presented major differences between the two different treatments at T2. CET participants presented a higher increase in resting-state BOLD connectivity (i.e., T2 > T1) in the DAN between the FEF and the precuneus [(*x*, *y*, *z*) = (−14, −46, 48), *t* = 4.28, *k* = 284], *p* < 0.005 uncorrected (whole-brain analysis), compared to TAU participants (Table 2, Fig. 3). In the SN, the CET group showed increased resting-state BOLD connectivity bilaterally from the ACC to the insula [(*x*, *y*, *z*) = (−32, −30, 12), *t* = 4.24, *k* = 70; (*x*, *y*, *z*) = (36, −30, 22), *t* = 3.75, *k* = 40] when compared to the TAU group (Table 3, Fig. 4). For this same network, the CET group displayed decreased resting-state BOLD connectivity bilaterally between the ACC and the putamen and globus pallidus [(*x*, *y*, *z*) = (14, 8, -6), *t* = 4.67, *k* = 64; (*x*, *y*, *z*) = (−16, 2, 0), *t* = 3.91, *k* = 40] in comparison with the TAU group (Fig. 5). There were further results from this comparison, including weakened functional connectivity within the ACC, with the supramarginal gyrus, and with the middle temporal gyrus (Table 4). The DMN results presented enhanced rsFC between the PCC and the precuneus, inferior parietal lobule, and angular gyrus (Table 5).

**Table 2:** Regions in the brain where increase in resting-state connectivity (i.e., T2 > T1) for the DAN with the Frontal Eye Field (FEF) as the seed was significantly higher in CET patients than in TAU patients

Side	Lobe	Brain areas	Brodmann area	Cluster size	MNI coordinates			*t*_maximum_
Left	Parietal	Precuneus; paracentral lobule	7, 5	284	−14	−46	48	4.28

Combined voxel-wise- (*p* < 0.005) and cluster-extent threshold (*k* ≥ 40 voxel) corresponding to *p*FWE < 0.05

*MNI* Montreal Neurological Institute

**Table 3:** Regions in the brain where resting-state connectivity (i.e., T2 > T1) for the SN with the anterior cingulate cortex (ACC) as the seed was significantly higher in CET patients than in TAU patients

Side	Lobe	Brain areas	Brodmann area	Cluster size	MNI coordinates			*t*_maximum_
Left	Sub-lobar	Insula; transverse temporal gyrus	13	70	−32	−30	12	4.24
Right	Sub-lobar	Insula	13	60	36	−30	22	3.75

Combined voxel-wise- (*p* < 0.005) and cluster-extent threshold (*k* ≥ 40 voxel) corresponding to *p*FWE < 0.05

*MNI* Montreal Neurological Institute

**Table 4:** Regions in the brain where the increase in resting-state connectivity (i.e., T2 > T1) for the SN with the ACC as the seed was significantly higher in TAU patients than in CET patients

Side	Lobe	Brain areas	Brodmann area	Cluster size	MNI coordinates			*t*_maximum_
Right	Sub-lobar	Putamen; globus pallidus		64	14	8	-6	4.67
Left and right	Sub-lobar	Corpus callosum; anterior cingulate cortex		91	−6	30	12	3.96
Left	Sub-lobar	Lateral globus pallidus; putamen		50	−16	2	0	3.91
Left	Parietal	Supramarginal gyrus; inferior parietal lobule	40	63	−46	−42	36	3.82
Right	Temporal	Middle temporal gyrus	21	48	68	−44	−4	3.43

Combined voxel-wise- (*p* < 0.005) and cluster-extent threshold (*k* ≥ 40 voxel) corresponding to *p*FWE < 0.05

*MNI* Montreal Neurological Institute

**Table 5:** Regions in the brain where resting-state connectivity (i.e., T2 > T1) for the DMN with the posterior cingulate cortex (PCC) as the seed was significantly higher in CET patients than in TAU patients

Side	Lobe	Brain areas	Brodmann area	Cluster size	MNI coordinates			*t*_maximum_
Left	Parietal	Inferior parietal lobule; precuneus; superior parietal lobule	7, 40	81	−30	−52	50	3.62
Right	Parietal	Angular gyrus; inferior parietal lobule	39	55	40	−64	36	3.57

Combined voxel-wise- (*p* < 0.005) and cluster-extent threshold (*k* ≥ 40 voxel), corresponding to *p*FWE < 0.05

*MNI* Montreal Neurological Institute

## Discussion

After the 3-week duration treatment, we could confirm our hypothesis by finding major differences in resting-state connectivity between CET participants and TAU participants. This study was designed to compare resting-state functional connectivity between CET and TAU in participants with AUD. Major differences in key regions related to cue-reactivity and salience processing were found. The main finding was increased functional connectivity in the SN between the ACC and the Insula in the CET group, while decreased functional connectivity was found between the ACC and the putamen and globus pallidus in the same group. Regarding the DAN, CET participants presented enhanced functional connectivity between the FEF and the precuneus after treatment. Also, the PCC showed increased rsFC with the precuneus in the DMN.

The precuneus has been related to consciousness due to its implication in the DMN and, more importantly, in many neuropsychiatric disorders that include impaired consciousness (Cavanna 2007). Its functions are suggested to be related to self-referential processes, autobiographic memory retrieval, and cognitive control (Cavanna 2007; Cavanna and Trimble 2006; Konova et al. 2013). The meta-analysis done by Konova et al. (2013) identified the precuneus, along with other regions, to be commonly activated by cognitive-based interventions that involve self-referential processing, cognitive control, and attention. Our study shows an increase of rsFC between the PCC and the precuneus after CET in the DMN. It is remarkable that also the FEF (from the DAN) displayed increased resting-state BOLD connectivity with the precuneus after CET. This increase in FC during the resting state with the precuneus is remarkable and could be of interest since it shows a pattern of increased connectivity between 3 important nodes—FEF–Precuneus–PCC

Previous studies have described an increase in fMRI response in the FEF area, one of the core nodes of the DAN, in front of reward-related visual stimuli (Chen et al. 2020; Serences 2008). Our results show an increase of rsFC between the FEF and the Precuneus after CET. This connectivity change is related to enhanced interaction between a visual and cue-reactive area of the brain, the FEF (Chen et al. 2020), with another that can integrate autobiographic memories and is related to decision-making, the precuneus (Cavanna 2007; Cavanna and Trimble 2006). Therefore, CET alters the functional connectivity between autobiographical and salience-detecting brain regions. This suggests that functional connectivity relates the saliency of visual cues with self-centered memories, but it should still be demonstrated whether these memories support or hinder abstinent behaviour. The changes in the DAN may support better regulation of the cue-reactivity exerted by the SN in the bottom-up regulation of attention. In AUD, the SN is overactivated, so the dynamic regulation between the DAN and the SN described by Vossel et al. (2014) is disrupted, bringing stronger salience effects in front of alcohol cues. This process ends in a flawed imbalance between bottom-up and top-down regulation of attention typical of substance use disorders (Zilverstand et al. 2018). Our results suggest CET might have an influence on this balance and further impact on the function of visuospatial attention guidance. Improved DAN connectivity can be related to better attentional and salience control in front of alcohol cues (Corbetta and Shulman 2002; Kincade et al. 2005).

The putamen and the globus pallidus displayed decreased functional connectivity in the SN from the ACC in the CET group. Both areas are part of the dorsal region of the striatum, and previous studies have related enhanced activation of the dorsal striatum in front of alcohol cues with elevated levels of alcohol consumption and dependence (Everitt and Robbins 2016; Vollstädt-Klein et al. 2010). Our results comparing AUD and HC showed higher resting-state BOLD connectivity within these regions in AUD. In agreement with these results, metabolic activity in the ACC and the striatum has also been reported to be higher in patients with AUD (Bralet et al. 2022). The ACC has been related to different decision-making and cognitive control processes (Menon and Uddin 2010) by inhibitory control in cognitive-based attention (Becker et al. 2018). This area was described to be stimulated by drug-related cues in several studies and different drug types and related to drug reward anticipation by encoding the motivational value of stimuli (Grüsser et al. 2004; Heinz et al. 2009; Schacht et al. 2013; Vollstädt-Klein et al. 2010). It is suggested to be the one selecting and evaluating external stimuli to propose the form of action to be implemented by the motor system accordingly (Bush et al. 2002). Since ACC collects environmental information and inputs, then processes the drug-related cues and relates them to the dorsal striatum, we suggest that this increase of activation in front of drug-related cues may cause the activation of the dorsal striatum which is the main responsible for craving and drug-seeking behaviour (Everitt and Robbins 2005; Müller-Oehring et al. 2015). Therefore, the decrease of resting-state connectivity between the ACC and the striatum after CET would protect the patient from excessive craving and drinking desires in front of alcohol-related cues by splitting this synchronic activity of both regions as a compensation mechanism. The ACC still receives the salient stimuli of environmental cues, but the striatum might be less sensitive to them because it is not receiving the same amount of stimulation as before the treatment. A decrease in resting-state connectivity from the ACC to the striatum may reflect an improvement of cue-reactivity control by compensation of aberrant AUD connectivity between these regions (Müller-Oehring et al. 2015; Schacht et al. 2013). Thus, in front of alcohol-related cues, the participant would not “feel” the urge to drink and could probably resist craving more easily.

Despite expecting an increase in functional connectivity with the IPL or the precuneus, we reported an increase in functional connectivity between the ACC and the insula, which rectifies one of the most described impairments in AUD. It has been widely described as a decrease in functional connectivity in the ACC and the insula in AUD (Everitt and Robbins 2016; Koob and Volkow 2016; Sullivan et al. 2013). Our results also reported this connectivity impairment in AUD participants when compared to HC. The insula has been repeatedly described as a region processing interoceptive information, mediating the internal state of the individual and the environmental inputs arriving (Eckert et al. 2009; Koob and Volkow 2016; Taylor et al. 2009). The region has been further implicated in salience awareness (Menon and Uddin 2010), being involved in interoceptive integration and emotional salience (Taylor et al. 2009). Since the insula is described as receiving multimodal sensory inputs, the decrease in functional connectivity between the insula and the ACC would reflect changes in sensory processing. Lower functional connectivity between these regions may reflect a reduction of the effect that alcoholic cues have on the participant’s emotional salience.

CET has been proposed as an inhibitory training that could facilitate the increase of cognitive control in the subjects undergoing it (Becker et al. 2018). It was proposed that new associations should be made by active inhibition and learning to restore undesired behaviours instead of unlearning or eliminating these previously established associations (Gass and Chandler 2013). The effect of CET in AUD has been analyzed in previous studies and demonstrated to have an impact on impaired cue-induced activation of several brain regions, like the ACC among others (Kiefer et al. 2015). Our results are in line with that study, showing decreased connectivity between the ACC and the dorsal striatum as a consequence of CET. Accordingly, the inhibitory training may have interfered in the functional connectivity synchronizing ACC and the striatum’s activation and therefore helped to obstruct the cue-reactivity processing in the brain before it turns into an overwhelming craving.

Becker et al. (2018), who reported also data from our sample, reported an increase in self-reported clinical outcome measures after CET. They found a positive correlation between increased ACC activation and the improvement of self-reported efficacy to abstain in CET participants. In that study, authors associated these changes with an elevated baseline reward sensitivity. However, it further suggested that brain functional connectivity changes can be related to a perceived improvement in self-efficacy.

In conclusion, our results, together with previous studies, improve our knowledge of the neural mechanisms underlying CET by providing fMRI evidence of its effect on resting-state connectivity. Nevertheless, we must keep in mind that our results are derived from a specific sample with patients abstinent for a determined time period. Results should be taken into consideration carefully because the impact of CET reported in this study may not last forever. Moreover, studies with more participants and better group demographics would help provide better statistical strength. Nowadays alcohol cues are present everywhere and abstinent patients must face different levels of exposure every day. Exposure to alcohol cues they were not really prepared for. CET prepares patients to face these situations they will repeatedly live in the near future, enhancing their ability to hold on to spontaneous alcohol consumption appetite through inhibition training. Because of this, CET includes the familiarisation of habitual scenarios where the patient will face different kinds of alcohol cues. A recent study reports habituation of the salience processing after repeated drug cue exposure in abstinent and treatment-engaged patients (Ekhtiari et al. 2021). Further studies are needed to see how long these effects last and to see if there is an impact on relapse rates and other clinical outcomes.

### Supplementary information

Below is the link to the electronic supplementary material.

Supplementary file1 (DOCX 51.4 KB)
