# Supplementary material for: Cue-exposure treatment influences resting-state functional connectivity—a randomized controlled fMRI study in alcohol use disorder
Source: Psychopharmacology (Berl). 2024 Jan 23;241(3):513–24. doi: 10.1007/s00213-024-06531-x (PMC10884177; doi:10.1007/s00213-024-06531-x)
Supplement: Supplementary file 1 — Supplementary file1 (DOCX 51.4 KB) [file 213_2024_6531_MOESM1_ESM.docx]

**Cue-Exposure Treatment influences resting-state functional connectivity – a randomized controlled fMRI study in Alcohol Use Disorder**

Àlvar Farré-Colomés^1^, Haoye Tan^1^, Sarah Gerhardt^1^, Martin Fungisai Gerchen^2,3,4^, Martina Kirsch^1^, Sabine Hoffmann^1^, Peter Kirsch^2,3,4^, Falk Kiefer^1,5,6^, Sabine Vollstädt-Klein^1,5^

^1^*Department of Addictive Behaviour and Addiction Medicine, Central Institute of Mental Health, Medical Faculty of Mannheim, Heidelberg University, 68159, Mannheim, Germany.*

^2^*Department of Clinical Psychology, Central Institute of Mental Health, Medical Faculty of Mannheim, Heidelberg University, 68159, Mannheim, Germany.*

^3^*Bernstein Center for Computational Neuroscience Heidelberg/Mannheim, 68159, Mannheim, Germany.*

^4^*Department of Psychology, Heidelberg University, 69117, Heidelberg, Germany.*

^5^*Mannheim Center for Translational Neurosciences (MCTN), Medical Faculty of Mannheim, Heidelberg University, 68159, Mannheim, Germany.*

^6^*Feuerlein Center on Translational Addiction Medicine, Heidelberg University, 69117, Heidelberg, Germany*

*
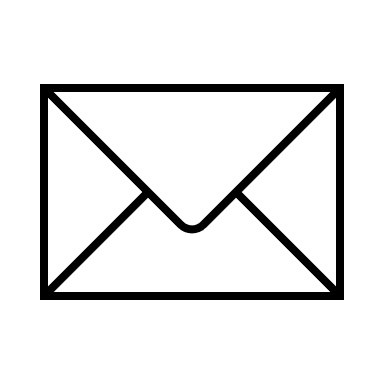
Corresponding author:* [*s.vollstaedt-klein@zi-mannheim.de*](mailto:s.vollstaedt-klein@zi-mannheim.de)

**Supplementary Results**

The comparison between treatment groups at T2 without SVC presented wider results. Increased connectivity in CET compared to TAU participants was reported towards the Precuneus and the Paracentral Lobule from the FEF in the DAN (Supplementary Table 2). Further, the CET group displayed reduced connectivity in the anterior PFC and OFC from the FEF in this same network (Supplementary Table 3). In the SN, a bilateral increase of connectivity between the Insula and the ACC in the CET group compared to the TAU group was reported (Supplementary Table 4). Instead, CET participants displayed decreased connectivity compared to TAU participants towards the Putamen and the ACC (Supplementary Table 5) after the treatment. Results regarding the DMN show increased BOLD signal in CET compared to TAU from PCC towards the Middle Frontal Gyrus and Precuneus (Supplementary Table 6). Decreased connectivity in CET compared to TAU was found between the PCC and the Superior Temporal Gyrus, Precentral Gyrus and Orbitofrontal Cortex (Supplementary Table 7).

A comparison between the HC and AUD groups (including CET + TAU participants) was performed at T1 (before treatment). In the DMN, the AUD group showed increased connectivity patterns bilaterally towards the Fusiform Gyrus, the Middle Occipital and Middle Temporal Gyri, and both the Caudate and the ACC from the PCC (Supplementary Table 8). Instead, decreased functional connectivity in the AUD group was reported to the Insula, SMG and Medial Frontal Gyrus, including the dorsolateral PFC, the medial PFC and the dACC (Supplementary Table 9). The analysis of the DAN showed increased connectivity in the AUD group between the FEF and the OFC (Supplementary Table 10), and decreased connectivity compared to the HC of the FEF with the ACC, the Middle Temporal Gyrus and Middle Occipital Gyrus, Broca’s Area, Calcarine Sulcus and Cuneus (Supplementary Table 11). Regarding the SN, AUD participants displayed increased connectivity only from FEF and the Caudate and Middle Frontal Gyrus (Supplementary Table 12), but they presented huge clusters representing decreased functional connectivity. These clusters included several regions: the Insula, Lentiform Nucleus, Thalamus, Cingulate Cortex, etc. All the clusters are displayed in Supplementary Table 13.

| **Supplementary Table 2:**  Regions in the brain where resting-state connectivity (i.e. T2 > T1) for the **DAN** with the Frontal Eye Fields (FEF) as seed was significantly higher in **CET** patients than in **TAU** patients. | | | | | | | | | | | | | | | | | | | | | | | |  |  |
| --- | --- | --- | --- | --- | --- | --- | --- | --- | --- | --- | --- | --- | --- | --- | --- | --- | --- | --- | --- | --- | --- | --- | --- | --- | --- |
| Side | | Lobe | | Brain Areas | | Brodmann Area | | Cluster Size | | MNI Coordinates | | | | | | | | | t_maximum_ | | | | |  |  |
| Left | | Parietal | | Precuneus  Paracentral Lobule | | 7, 5 | | 413 | | -14 | | -46 | | | 48 | | | | | 4.28 | | | | | |
| Left | | Frontal | | Paracentral Lobule | | 6 | | 61 | | -10 | | -26 | | | 50 | | | | | 4.00 | | | | | |
| Right | | Parietal | | Precuneus  Paracentral Lobule | | 7, 5 | | 62 | | 16 | | -44 | | | 52 | | | | | 3.86 | | | | | |
| *Note.* MNI = Montreal Neurological Institute. Combined voxel-wise- [*P* < 0.005] and cluster-extent threshold [*k* ≥ 40 voxel], corresponding to *p*FWE < 0.05 | | | | | | | | | | | | | | | | | | | | | |  |  |  |  |
| **Supplementary Table 3:**  Regions in the brain where resting-state connectivity (i.e. T2 > T1) for the **DAN** with the FEF as seed was significantly higher in **TAU** patients than in **CET** patients. | | | | | | | | | | | | | | | | | | | | | | | |  |  |
| Side | | Lobe | | Brain Areas | | Brodmann Area | | Cluster Size | | MNI Coordinates | | | | | | | | | t_maximum_ | | | | |  |  |
| Right | | Frontal | | Middle Frontal Gyrus | | 11 | | 124 | | 26 | | 42 | | | 0 | | | | | 4.46 | | | | | |
| Left | | Frontal | | Middle Frontal Gyrus  Inferior Frontal Gyrus | | 10 | | 118 | | -40 | | 52 | | | 6 | | | | | 3.94 | | | | | |
| Left | | Frontal | | Middle Frontal Gyrus | | - | | 76 | | -26 | | 38 | | | -2 | | | | | 3.54 | | | | | |
| *Note.* MNI = Montreal Neurological Institute. Combined voxel-wise- [*P* < 0.005] and cluster-extent threshold [*k* ≥ 40 voxel], corresponding to *p*FWE < 0.05 | | | | | | | | | | | | | | | | | | | | | |  |  |  |  |
| **Supplementary Table 4:**  Regions in the brain where resting-state connectivity (i.e. T2 > T1) for the **SN** with the Anterior Cingulate Cortex (ACC) as seed was significantly higher in **CET** patients than in **TAU** patient. | | | | | | | | | | | | | | | | | | | | | | | |  |  |
| Side | | Lobe | | Brain Areas | | Brodmann Area | | Cluster Size | | MNI Coordinates | | | | | | | | | t_maximum_ | | | | |  |  |
| Left | | Sub-Lobar | | Insula | | 13 | | 184 | | -32 | | -30 | | | 10 | | | | | 4.43 | | | | | |
| Right | | Sub-Lobar | | Insula | | 13 | | 173 | | 30 | | -10 | | | 18 | | | | | 3.77 | | | | | |
| *Note.* MNI = Montreal Neurological Institute. Combined voxel-wise- [*P* < 0.005] and cluster-extent threshold [*k* ≥ 40 voxel], corresponding to *p*FWE < 0.05 | | | | | | | | | | | | | | | | | | | | | |  |  |  |  |
| **Supplementary Table 5:**  Regions in the brain where resting-state connectivity (i.e. T2 > T1) for the **SN** with the ACC as seed was significantly higher in **TAU** patients than in **CET** patients. | | | | | | | | | | | | | | | | | | | | | | | |  |  |
| Side | | Lobe | | Brain Areas | | Brodmann Area | | Cluster Size | | MNI Coordinates | | | | | | | | | t_maximum_ | | | | |  |  |
| Right | | Sub-Lobar | | Lentiform Nucleus  Putamen | | - | | 129 | | 14 | | | 8 | | | -6 | | | | 4.67 | | | | | |
| Right | | Frontal | | Middle Frontal Gyrus | | 9 | | 43 | | 48 | | | 32 | | | 40 | | | | 4.42 | | | | | |
| Left | | Sub-Lobar | | Lentiform Nucleus  Putamen | | - | | 140 | | -18 | | | 2 | | | 2 | | | | 4.01 | | | | | |
| Left | | Sub-Lobar | | Anterior Cingulate Cortex | | 24 | | 149 | | -8 | | | 28 | | | 14 | | | | 3.98 | | | | | |
| Left | | Parietal | | Supramarginal Gyrus  Inferior Parietal Lobule | | 40 | | 138 | | -46 | | | -42 | | | 36 | | | | 3.82 | | | | | |
| Right | | Frontal | | Middle Frontal Gyrus  Superior Frontal Gyrus | | 10 | | 70 | | 12 | | | 54 | | | 2 | | | | 3.60 | | | | | |
| Right | | Temporal | | Hippocampus  Fusiform Gyrus | | 20 | | 46 | | 42 | | | -12 | | | -26 | | | | 3.50 | | | | | |
| Right | | Temporal | | Middle Temporal Gyrus | | 21 | | 81 | | 68 | | | -44 | | | -4 | | | | 3.43 | | | | | |
| Left | | Frontal | | Middle Frontal Gyrus | | 9, 8 | | 45 | | -36 | | | 28 | | | 42 | | | | 3.41 | | | | | |
| *Note.* MNI = Montreal Neurological Institute. Combined voxel-wise- [*P* < 0.005] and cluster-extent threshold [*k* ≥ 40 voxel], corresponding to *p*FWE < 0.05 | | | | | | | | | | | | | | | | | | | | | |  |  |  |  |
| **Supplementary Table 6:**  Regions in the brain where resting-state connectivity (i.e. T2 > T1) for the **DMN** with the PCC as seed was significantly higher in **CET** patients than in **TAU** patients. | | | | | | | | | | | | | | | | | | | | | | | |  |  |
| Side | | Lobe | | Brain Areas | | Brodmann Area | | Cluster Size | | MNI Coordinates | | | | | | | | | t_maximum_ | | | | |  |  |
| Left | | Frontal | | Middle Frontal Gyrus | | - | | 86 | | -24 | | 12 | | | 38 | | | | | 3.95 | | | | | |
| Left | | Parietal | | Inferior Parietal Lobule  Superior Parietal Lobule  Precuneus | | 7, 40 | | 145 | | -30 | | -52 | | | 50 | | | | | 3.62 | | | | | |
| Right | | Parietal | | Inferior Parietal Lobule  Angular Gyrus | | 39 | | 111 | | 40 | | -64 | | | 36 | | | | | 3.57 | | | | | |
| Left | | Parietal | | Precuneus | | 7 | | 40 | | -20 | | -72 | | | 52 | | | | | 3.39 | | | | | |
| *Note.* MNI = Montreal Neurological Institute. Combined voxel-wise- [*P* < 0.005] and cluster-extent threshold [*k* ≥ 40 voxel], corresponding to *p*FWE < 0.05 | | | | | | | | | | | | | | | | | | | | | |  |  |  |  |
| **Supplementary Table 7:**  Regions in the brain where resting-state connectivity (i.e. T2 > T1) for the **DMN** with the PCC as seed was significantly higher in **TAU** patients than in **CET** patients. | | | | | | | | | | | | | | | | | | | | | | | |  |  |
| Side | | Lobe | | Brain Areas | | Brodmann Area | | Cluster Size | | MNI Coordinates | | | | | | | | | t_maximum_ | | | | |  |  |
| Right | | Temporal | | Superior Temporal Gyrus | | 22 | | 62 | | 42 | | -28 | | | 4 | | | | | 4.01 | | | | | |
| Right | | Frontal | | Precentral Gyrus  Postcentral Gyrus | | 3, 4 | | 44 | | 48 | | -18 | | | 40 | | | | | 3.35 | | | | | |
| Right | | Frontal | | Inferior Frontal Gyrus  Orbitofrontal Cortex | | 47 | | 54 | | 50 | | 26 | | | -10 | | | | | 3.26 | | | | | |
| *Note.* MNI = Montreal Neurological Institute. Combined voxel-wise- [*P* < 0.005] and cluster-extent threshold [*k* ≥ 40 voxel], corresponding to *p*FWE < 0.05 | | | | | | | | | | | | | | | | | | | | | |  |  |  |  |
| **Supplementary Table 8:**  Regions in the brain where resting-state connectivity for the **DMN** with the PCC as seed was significantly higher in **AUD** patients than in **HC** before the treatment (T1). | | | | | | | | | | | | | | | | | | | | | | | |  |  |
| Side | | Lobe | | Brain Areas | | Brodmann Area | | Cluster Size | | MNI Coordinates | | | | | | | | t_maximum_ | | | | | |  |  |
| Righ | | Temporal | | Fusiform Gyrus  Middle Temporal Gyrus  Inferior Temporal Gyrus | | 20, 36, 21 | | 369 | | 46 | | -14 | | | -20 | | | | 5.09 | | | | | | |
| Right | | Frontal | | Caudate  ACC | | - | | 99 | | 18 | | 30 | | | 2 | | | | 4.33 | | | | | | |
| Right | | Occipital | | Middle Occipital Gyrus  Inferior Occiptal Gyrus  Fusiform Gyrus | | 19 | | 642 | | 44 | | -66 | | | -14 | | | | 4.15 | | | | | | |
| Righ | | Sub-Lobar | | Fusiform Gyrus  Middle Temporal Gyrus  Parahippocampal Gyrus | | - | | 176 | | 32 | | -2 | | | -42 | | | | 4.03 | | | | | | |
| Left | | Temporal | | Fusiform Gyrus | | 20 | | 87 | | -42 | | -30 | | | -22 | | | | 3.90 | | | | | | |
| Right | | Temporal | | Superior Temporal Gyrus | | 41 | | 66 | | 44 | | -40 | | | 14 | | | | 3.70 | | | | | | |
| Left | | Occipital | | Middle Occipital Gyrus  Middle Temporal Gyrus | | 19, 37 | | 582 | | -38 | | -80 | | | 4 | | | | 3.59 | | | | | | |
| Right | | Temporal | | Middle Frontal Gyrus  Orbitofrontal Cortex | | 37 | | 68 | | 50 | | -54 | | | -24 | | | | 3.48 | | | | | | |
| *Note.* MNI = Montreal Neurological Institute. Combined voxel-wise- [*P* < 0.005] and cluster-extent threshold [*k* ≥ 40 voxel], corresponding to *p*FWE < 0.05 | | | | | | | | | | | | | | | | | | | | | |  |  |  |  |
| **Supplementary Table 9:**  Regions in the brain where resting-state connectivity for the **DMN** with the PCC as seed was significantly higher in **HC** than in **AUD** patients before the treatment (T1). | | | | | | | | | | | | | | | | | | | | | | | |  |  |
| Side | | Lobe | | Brain Areas | | Brodmann Area | | Cluster Size | | MNI Coordinates | | | | | | | | t_maximum_ | | | | | |  |  |
| Left | | Frontal | | Middle Frontal Gyrus  ACC | | 9, 32 | | 761 | | 0 | | 38 | | | 38 | | | | 5.21 | | | | | | |
| Left | | Frontal | | Middle Frontal Gyrus  Superior Frontal Gyrus | | 6, 8, 9, 10 | | 1410 | | -32 | | 26 | | | 32 | | | | 4.73 | | | | | | |
| Left | | Sub-Lobar | | Insula | | - | | 107 | | -32 | | 0 | | | 8 | | | | 3.70 | | | | | | |
| Right | | Parietal | | Supramarginal Gyrus | | 40 | | 120 | | 62 | | -44 | | | 38 | | | | 3.66 | | | | | | |
| Right | | Frontal | | Middle Frontal Gyrus | | 9 | | 90 | | 44 | | 28 | | | 34 | | | | 3.48 | | | | | | |
| Right | | Sub-Lobar | | ACC | | 24 | | 86 | | 8 | | 8 | | | 30 | | | | 3.42 | | | | | | |
| Right | | Frontal | | Middle Frontal Gyrus | | 10 | | 226 | | 30 | | 56 | | | 10 | | | | 3.39 | | | | | | |
| *Note.* MNI = Montreal Neurological Institute. Combined voxel-wise- [*P* < 0.005] and cluster-extent threshold [*k* ≥ 40 voxel], corresponding to *p*FWE < 0.05 | | | | | | | | | | | | | | | | | | | | | |  |  |  |  |
| **Supplementary Table 10:**  Regions in the brain where resting-state connectivity for the **DAN** with the FEF as seed was significantly higher in **AUD** patients than in **HC** before the treatment (T1). | | | | | | | | | | | | | | | | | | | | | | | |  |  |
| Side | | Lobe | | Brain Areas | | Brodmann Area | | Cluster Size | | MNI Coordinates | | | | | | | | t_maximum_ | | | | | |  |  |
| Left | | Frontal | | Orbitofrontal Cortex | | 47 | | 68 | | -36 | | 32 | | | -18 | | | | 3.33 | | | | | | |
| *Note.* MNI = Montreal Neurological Institute. Combined voxel-wise- [*P* < 0.005] and cluster-extent threshold [*k* ≥ 40 voxel], corresponding to *p*FWE < 0.05 | | | | | | | | | | | | | | | | | | | | | |  |  |  |  |
| **Supplementary Table 11:**  Regions in the brain where resting-state connectivity for the **DAN** with the FEF as seed was significantly higher in **HC** than in **AUD** patients before the treatment (T1). | | | | | | | | | | | | | | | | | | | | | | | |  |  |
| Side | | Lobe | | Brain Areas | | Brodmann Area | | Cluster Size | | MNI Coordinates | | | | | | | | t_maximum_ | | | | | |  |  |
| Left | | Sub-Lobar | | ACC | | 24 | | 254 | | -2 | | 26 | | | 0 | | | | 4.37 | | | | | | |
| Left | | Occipital | | Middle Temporal Gyrus  Middle Occipital Gyrus | | 39 | | 318 | | -36 | | -76 | | | 14 | | | | 4.05 | | | | | | |
| Right | | Frontal | | Inferior Frontal Gyrus | | 45 | | 145 | | 52 | | 24 | | | 6 | | | | 3.73 | | | | | | |
| Right | | Temporal | | Calcarine Sulcus  Cuneus | | 17, 31 | | 149 | | 26 | | -68 | | | 22 | | | | 3.61 | | | | | | |
| Right | | Temporal | | Middle Temporal Gyrus | | 39 | | 257 | | 46 | | -70 | | | 10 | | | | 3.59 | | | | | | |
| Left | | Occipital | | Cuneus  Lingual Gyrus | | 30, 23 | | 345 | | -14 | | -72 | | | 4 | | | | 3.57 | | | | | | |
| Right | | Frontal | | Superior Frontal Gyrus  Middle Frontal Gyrus | | 6 | | 71 | | 24 | | -8 | | | 74 | | | | 3.51 | | | | | | |
| Right | | Temporal | | Superior Temporal Gyrus  Middle Temporal Gyrus | | 22, 42 | | 145 | | 64 | | -28 | | | 8 | | | | 3.23 | | | | | | |
| *Note.* MNI = Montreal Neurological Institute. Combined voxel-wise- [*P* < 0.005] and cluster-extent threshold [*k* ≥ 40 voxel], corresponding to *p*FWE < 0.05 | | | | | | | | | | | | | | | | | | | | | |  |  |  |  |
| **Supplementary Table 12:**  Regions in the brain where resting-state connectivity for the **SN** with the ACC as seed was significantly higher in **AUD** patients than in **HC** before the treatment (T1). | | | | | | | | | | | | | | | | | | | | | | | |  |  |
| Side | | Lobe | | Brain Areas | | Brodmann Area | | Cluster Size | | MNI Coordinates | | | | | | | | t_maximum_ | | | | | |  |  |
| Right | | Frontal | | Caudate | | - | | 111 | | 20 | | 30 | | | 2 | | | | 4.13 | | | | | | |
| Left | | Sub-Lobar | | Caudate | | - | | 75 | | -12 | | 22 | | | 14 | | | | 4.10 | | | | | | |
| *Note.* MNI = Montreal Neurological Institute. Combined voxel-wise- [*P* < 0.005] and cluster-extent threshold [*k* ≥ 40 voxel], corresponding to *p*FWE < 0.05 | | | | | | | | | | | | | | | | | | | | | |  |  |  |  |
| **Supplementary Table 13:**  Regions in the brain where resting-state connectivity for the **SN** with the ACC as seed was significantly higher in **HC** than in **AUD** patients before the treatment (T1). | | | | | | | | | | | | | | | | | | | | | | | |  |  |
| Side | | Lobe | | Brain Areas | | Brodmann Area | | Cluster Size | | MNI Coordinates | | | | | | | | t_maximum_ | | | | | |  |  |
| Left | | Sub-Lobar | | Precentral Gyrus  Insula  Superior Temporal Gyrus  Lentiform Nucleus | | 13, 22, 6 | | 995 | | -42 | | -14 | | | 6 | | | | 4.74 | | | | | | |
| Right | | Occipital | | Cuneus  Calcarine Sulcus  Middle Occipital Gyrus | | 18, 19 | | 684 | | 26 | | -94 | | | 26 | | | | 4.50 | | | | | | |
| Right | | Sub-Lobar | | Thalamus  Lentiform Nucleus  Putamen  Amigdala  Parahippocampal Gyrus | | - | | 1132 | | 20 | | -2 | | | -12 | | | | 4.44 | | | | | | |
| Right | | Sub-Lobar | | Superior Temporal Gyrus  Insula  Precentral Gyrus  Middle Temporal Gyrus | | 22 | | 1461 | | 44 | | -18 | | | -4 | | | | 4.38 | | | | | | |
| Left | | Sub-Lobar | | Cingulate Gyrus | | 24 | | 192 | | -4 | | -10 | | | 40 | | | | 4.16 | | | | | | |
| Left | | Temporal | | Middle Temporal Gyrus  Superior Temporal Gyrus | | 21, 22, 38 | | 443 | | -60 | | -10 | | | -12 | | | | 4.13 | | | | | | |
| Right | | Frontal | | Inferior Frontal Gyrus | | 45, 46 | | 163 | | 48 | | 40 | | | 4 | | | | 4.00 | | | | | | |
| Right | | Sub-Lobar | | Insula | |  | | 87 | | 30 | | 16 | | | 18 | | | | 3.99 | | | | | | |
| Left | | Frontal | | Middle Frontal Gyrus  Superior Frontal Gyrus | | 10 | | 119 | | -4 | | 64 | | | 20 | | | | 3.58 | | | | | | |
| Right | | Sub-Lobar | | Posterior Cingulate Cortex  Calcarine Sulcus | | 23, 30 | | 204 | | 6 | | -64 | | | 14 | | | | 3.49 | | | | | | |
| Right | | Occipital | | Lingual Gyrus | | 18 | | 121 | | 8 | | -88 | | | -14 | | | | 3.48 | | | | | | |
| Right | | Sub-Lobar | | Parahippocampal Gyrus  Fusiform Gyrus | | 36, 20 | | 167 | | 34 | | -30 | | | -16 | | | | 3.48 | | | | | | |
| Right | | Temporal | | Superior Temporal Gyrus  Insula  Inferior Temporal Gyrus | | 38, 13 | | 200 | | 38 | | 10 | | | -16 | | | | 3.37 | | | | | | |
| Left | | Sub-Lobar | | Precuneus  Posterior Cingulate Cortex | | 31, 30 | | 144 | | -4 | | -58 | | | 26 | | | | 3.35 | | | | | | |
| *Note.* MNI = Montreal Neurological Institute. Combined voxel-wise- [*P* < 0.005] and cluster-extent threshold [*k* ≥ 40 voxel], corresponding to *p*FWE < 0.05 | | | | | | | | | | | | | | | | | | | | | |  |  |  |  |
